# Antifungal Activity of Biocontrol Agents In Vitro and Potential Application to Reduce Mycotoxins (Aflatoxin B1 and Ochratoxin A)

**DOI:** 10.3390/toxins13110752

**Published:** 2021-10-23

**Authors:** Francisco Illueca, Pilar Vila-Donat, Jorge Calpe, Carlos Luz, Giuseppe Meca, Juan M. Quiles

**Affiliations:** Laboratory of Food Chemistry and Toxicology, Faculty of Pharmacy, University of Valencia, Av. Vicent Andrés Estellés s/n, 46100 Burjassot, Spain; franima3@alumni.uv.es (F.I.); jorge.calpe@uv.es (J.C.); carlos.luz@uv.es (C.L.); giuseppe.meca@uv.es (G.M.); Juan.Quiles@uv.es (J.M.Q.)

**Keywords:** biocontrol agents, antifungal, mycotoxins, reduction, in vitro, bio-preservation

## Abstract

Food bio-preservatives are requested as substituents of chemical pesticides in food. The aim of this study was to carry out a screening of twenty biocontrol agents (BCAs) for their potential fungicidal activity in vitro. Twenty BCAs were tested against ten pathogenic fungi. Some of the cell-free supernatants (CFS) tested showed in vitro antifungal activity versus pathogenic fungi. The highest fungicidal activity was observed in the fermented CFS of *Paenibacillus chibensis* CECT 375, *Bacillus amyloliquefaciens* CECT 493, and *Pantoea agglomerans* CECT 850, which showed a minimum inhibitory concentration (MIC) and minimum fungicidal concentration (MFC) values of 125 and 250 g/L, respectively. The compounds responsible for the antifungal activity, such as organic and phenolic acids, were determined. Lactic acid, acetic acid, benzoic acid, and phenyllactic acid among others can be related to antifungal activity. HPLC-MS/MS analysis showed a reduction of ochratoxin A (OTA) and aflatoxin B_1_ (AFB_1_) up to 26% (*Paenibacillus alvei* CECT 2) and 55% (*Paenibacillus polymyxa* CECT 155), respectively. The present study prompts that metabolism products of BCAs are propitious for the bioconservation of food, due to their ability to reduce the proliferation of mycotoxigenic fungi and mycotoxins production.

## 1. Introduction

Fungi contamination remains a significant issue for the food industry due to their ability to degrade food and produce mycotoxins [1]. Fungal contamination of crops occurs at various stages during pre-harvest and postharvest, producing economic losses for farming and health problems in animals and humans [2,3,4]. It is estimated that about 10% of world basic staples are lost due to fungal contamination [5].

From the toxicological perspective, the most significant mycotoxins are aflatoxins. Aflatoxins are toxic metabolites mainly produced by *Aspergillus flavus* and *Aspergillus parasiticus.* Among them, aflatoxin B_1_ (AFB_1_) is the most worrisome. It has been classified as a group 1 compound, due to its carcinogenic effect, by the International Agency for Research on Cancer (IARC) [6]. In long-term exposure, this mycotoxin is associated to liver carcinoma in humans [7,8]. Another concerning mycotoxin is ochratoxin A (OTA), which is a nephrotoxic substance. It also possesses teratogenic, immunotoxic, and possibly neurotoxic properties. IARC classifies OTA as a possibly carcinogenic compound in humans (group 2B) [9]. These mycotoxins are still present in high and low levels in food and feed, causing a diversity of adverse effects, from acute to chronic, both in humans and animals [4,10].

The use of synthetic fungicides has been the most common tool to fight this type of food deterioration, but its use has certain objections [11]. Pesticides have helped reduce crop loss caused by several infections and pests. However, constant usage of the same chemicals causes undesirable effects, such as pesticide resistance and environmental degradation, urging the need for alternative strategies that can protect crop health sustainably. In addition to the above mentioned, the consumer and public authorities demand the reduction in the use of chemical pesticides, encouraging us toward environmentally safe and sustainable substances for food preservation [12].

Scientific evidence related to the biological control indicates the ability of microorganisms such as yeast, bacteria, and fungi to mitigate mycotoxins by preventing their presence in animal feed and food of animal origin [10]. Microbial decontamination by BCAs is becoming a trust strategy contributing to food safety. Some bacteria genera such as *Bacillus, Pseudomonas, Stenotrophomonas,* and *Streptomyces* and fungi belonging to the genus *Trichoderma* act against a wide range of plant pathogens in an environmentally sustainable manner [13]. Several studies have screened the efficacy of potential microorganisms for mycotoxin control; however, the vast majority of investigated bacteria were mainly lactic acid bacteria (LABs) [1,14,15,16,17,18,19]. Non-lactic acid bacteria such as *Bacillus* spp. can also inhibit fungi growth and mycotoxin production [20]. Recently, Calvo et al. (2017) showed that *Bacillus amyloliquefaciens BUZ-14* produce several bioactive compounds with high fungicidal activity such as lipopeptides and several polyketides, and their results indicated that *B. amyloliquefaciens* was promising BCA for postharvest fruit diseases [20]. BCAs act on competitive exclusion of mycotoxigenic pathogens, producing metabolites that may inhibit the colonization of crops and reduce the capability of mycotoxin production [13].

Given the rising interest in the use of natural microorganisms as an alternative to synthetic pesticides and the potential of BCAs, the aims of the present work were: (a) to evaluate the antifungal activity of twenty BCAs (bacteria, yeast, and fungi) against ten different fungal pathogenic species (b) to investigate the phenolic and organic acids produced by these bioactive strains and (c) to analyze the mycotoxin AFB_1_ and OTA reduction in vitro. The use of different BCAs could represent an essential food preservation strategy as well as become the main alternative to chemical preservatives in food.

## 2. Results and Discussion

### 2.1. In Vitro Antifungal Activity of Biocontrol Strains

The overlay assay showed a high antifungal activity of twenty isolated BCAs versus *Penicillium, Aspergillus, Fusarium,* and *Alternaria* species (ten in total) expressed as percentage of inhibition after seven days with respect to the control. The strains *Paenibacillus polymyxa* CECT 155 and *Metschnikowia pulcherrima* CECT 10408 showed percentage of inhibition (up to 20%) for all pathogens tested, even reaching 50% of inhibition in most cases with the exception of *Aspergillus flavus* (ITEM 8111) (Table 1). *P. polymyxa* CECT 155, *Pantoea agglomerans* CECT 850, *M. pulcherrima* CECT 1691, and CECT 10408 evidenced the highest antifungal activities against almost all *Fusarium* pathogenic fungi tested. *P. polymyxa* CECT 155 showed percentage inhibition of 50.5%, 48.2%, 40.3%, and 46% for *Fusarium graminerarum*, *Fusarium poae*, *Fusarium verticilloides,* and *Fusarium langsethiae*, respectively. *P. agglomerans* inhibited 47.4% of *Fusarium sporotrichoides*, whereas strains of *M. pulcherrima* (CECT 1691 and CECT 10408) showed inhibition of 50.4% and 50.1% for *Fusarium proliferatum*, respectively (Table 1).

**Figure 1:**
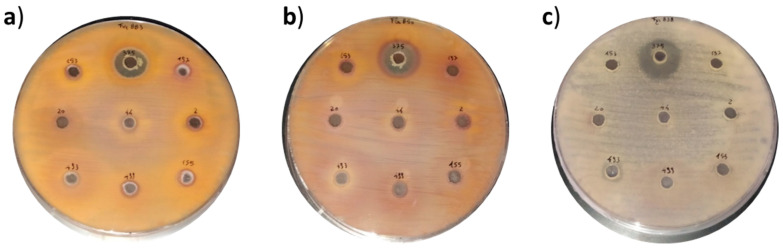
Example of antifungal activity of *Paenibacillus chibensis* CECT 375 against: (**a**) *Fusarium verticilloides* ITEM 12044, (**b**) *Fusarium graminearum* ITEM 126, and (**c**) *Fusarium langsethiae* ITEM 11031 determined by the diffusion agar method.

On the other hand, some of the CFS (cell-free supernatants) of biocontrol microorganisms tested proved antifungal activity against pathogenic fungi in the solid medium diffusion agar test (Table 2). The highest antifungal activity was detected in the CFS of *Paenibacillius chibensis* and *P. agglomerans*, which inhibited the growth of almost all the pathogens tested, and *Bacillus amyloliquefaciens*, which inhibited the growth of *F. Poae* and *F. graminearum* by the diffusion agar method (Table 2). The antifungal capacity of *P. chibensis* against *Fusarium verticilloides*, *F. graminearum*, and *F. langsethiae* was proved, as can be clearly seen in Figure 1. In accordance with these results, other authors evidenced the antifungal activity of bacteria against different fungi species by using the diffusion agar method [1,16,19] and overlay method [17]. However, none of these studies used such selection of BCAs including non-lactic acid bacteria, yeast, and fungi. With the aim to quantify the potential antifungal activity of the three bioactive CFS (*P. chibensis, P. agglomerans*, and *B. amyloliquefaciens*), the minimum inhibitory concentration (MIC) and minimum fungicidal concentration (MFC) values were determined. The results of MIC-MFC tests are described in Table 3. In line with qualitative results, the three CFS tested showed inhibitory activity reaching MIC-MFC values from 125 to 250 g/L against almost all the fungi tested (*Penicillium* spp., *Aspergillus* spp., *Fusarium* spp., and *Alternaria* spp.). The three biocontrol strains tested (*P. chibensis*, *B. amyloliquefaciens,* and *P. agglomerans*) showed MIC and CFS values of 125 and 250 g/L, respectively, against *F. langsethiae*. Meanwhile, the CFS of strain PA850 showed MIC and CFS values (125 and 250 g/L, respectively) against *Penicillium and Alternaria* strains. *P. chibensis* showed such values against *F. graminearum* and *A. flavus*. These results were in accordance with Luz et al. (2020) [16], who evidenced that the *Fusarium* genus was the most sensitive specie to the bioactive compounds present in the fermented CFS of *Lactobacillus plantarum*. In the same work, the authors showed MFC values of 250 g/L from LABs against *Aspergillus* [16]. In the same line, Izzo et al. (2020) investigated the inhibitory effect of sweet whey fermented by *Lactobacillus plantarum* strains against fungal growth (*Fusarium* and *Aspergillus*), and the results of the MIC–MFC varied between 1.95 and 250 g/L, whereas the value of MFC was in the range of 4 to 250 g/L [12]. Hence, MIC and MFC values obtained in this work are similar comparing with values previously reported in which LABs were tested to increase the shelf life of food [12,16,19]. These results suggest the possible use of BCAs as a source of new preservatives of natural origin to incorporate in food matrices for the purpose of improving the shelf life. However, due to the Good Hygienic and Manufacturing Practices required for food production and their low microbial load, the use of concentrations significantly below the MIC and MFC values could also result in a significant increase in shelf life.

In contrast, other studies, such as Rizzello, et al. (2011) [21], evidenced lower MIC values (2.5–15.2 g/L) for the water-soluble extract from sourdough fermented by LABs when proved against *Penicillium* spp.

### 2.2. Identification of Antifungal Compounds in CFS

In this study, two organic acids and seven phenolic acids were identified in CFS fermented by twenty and nine biocontrol strains, respectively (Table 4 and Table 5). These compounds were determined by HPLC-qTOF-MS. The organic acids in lyophilized CFS fermented by BCAs are indicated in Table 4. Two organic acids were determined: lactic acid and acetic acid. All isolated BCAs produced lactic acid, reaching concentrations from 129 to 3491 mg/L. The BCAs with the highest lactic acid production were *Bacillus thuringiensis* (3491 mg/L), *P. polymyxa* CECT 155 (3372 mg/L), *P. polymyxa* CECT 153 (2622 mg/L), *Bacillus subtilis* (2553 mg/L), and *Bacillus megaterium* (2350 mg/L). The production of acetic acid (203–1873 mg/L) was determined only in five CFS, the highest concentration produced being *P. polymyxa* CECT 153 and *P. polymyxa* CECT 155 (1873 mg/L and 1271 mg/L, respectively). Table 5 lists the nine fermented CFS in which phenolic acids were detected. These phenolic acids detected and quantified in the CFS were DL-3-phenyl lactic acid (PLA), benzoic acid, 1-2-dydroxybenzene, hydroxycinnamic acid, p-coumaric acid, 3-4-dihydroxycinnamic acid, and protocatechuic acid. Some of these compounds have been previously reported as antifungal agents produced by LABs [18]. Benzoic acid was quantified in each CFS tested, and its concentration range was 0.004–0.067 g/L. The highest concentrations of benzoic acid were detected in *P. alvei* and *P. polymyxa* CECT 153 (0.067 and 0.060 g/L, respectively).

PLA was detected only in five CFS, and concentrations ranged from 0.058 to 0.844 g/L, among which the *B. thuringiensis* strain was the highest producer of PLA. In addition, 1-2-dyhdroxybenzene was quantified only in two CFS (*B. amyloliquefaciens* and *P. chibensis*), with values of 0.03–0.04 g/L, respectively. These results are in line with Rodriguez et al. (2008), who noticed the presence of 1-2-dyhdroxybenzene generated by enzymes of *Lactobacillus plantarum* [22]. The fact that *P. polymyxa* CECT 155 and *P. chibensis* were the only CFS that produced dydroxyhydrocinnamic (0.022 ± 0.06 g/L) and protocatechuic acid (0.008 ± 0.01 g/L) respectively, should be highlighted.

Our results pointed out that strains of *P. polymyxa* CECT 153, *P. polymyxa* CECT 155, *P. chibensis*, *B. amyloliquefaciens*, and *P. alvei* showed the highest capacity to produce organic and phenolic compounds. Like organic acid production, the phenolic acids data were associated with the observed antifungal activity of CFS. In fact, previous authors evidenced synergism between lactic acid and acetic acid [23], and between these and PLA regarding the potential antifungal activity [24]. 

Moreover, some of these phenolic compounds with antifungal activity, above described, have been linked to the ability to prevent mycotoxin production [1,19,25,26].

### 2.3. Antimycotoxigenic Activity of CFS

Along with evaluating the qualitative and quantitative antifungal activity of CFS, the ability to reduce mycotoxins was investigated. The antimycotoxigenic activity was evaluated by using 20 biocontrol strains against AFB_1_ (1 mg/L) and OTA (1 mg/L) after 48 h of exposure (Table 6). To our knowledge, this is the first work in which the antimycotoxigenic activity of 20 different BCAs against ten pathogens has been investigated. 

As evidenced in Table 6, 11 out of 20 and 14 out of 20 BCAs tested showed AFB_1_ and OTA reduction above 10%. The reduction of AFB_1_ was in the range of 1.2 to 55%, and the reduction percentage of OTA ranging from 4.7 to 26.5%*. P. polymyxa* CECT 155 showed the highest reduction (55%) of AFB_1_ after 48 h of exposure. These values are slightly higher than those found in other studies testing biocontrol strains. Topcu et al. (2010) tested some non-lactic acid bacteria, specifically probiotic *Enterococcus faecium* M74 and EF031 strains, and observed a reduction of the AFB_1_ content of aqueous solution by 19–38% [27]. However, other authors noticed that a *Bacillus subtilis* strain decreased the AFB_1_ level of contaminated feed and food by 60–95% [28,29]. These differences may be related to the type of biocontrol strain, days of exposure, and the presence or absence of a food matrix.

Moreover, in our study, *P. alvei* showed the highest reduction of OTA (26.5%) after 48 h of exposure. Similar results were obtained by Shukla et al. (2018) who isolated *Bacillus subtilis* KU-153 from Kimchi (a traditional Korean fermented food) and investigated its ability to lessen OTA content in culture medium. They observed a reduction of OTA content in viable cells by 22%. However, *B. subtilis* heat-treated cells proved a higher OTA reduction (45%) [30]. 

As previously mentioned, these biocontrol strains were among the great producers of phenolic compounds; hence, the production of metabolites with antifungal activity may explain their anti-mycotoxigenic activity.

The present results suggested that metabolism products of BCAs, due to their potential to minimize the growth of mycotoxigenic fungi and the production of the mycotoxins, could be promising for the bioconservation of food.

## 3. Conclusions

In this study, twenty different BCAs were tested against ten mycotoxigenic fungi. In vitro experiments proved that CFS fermented by isolated BCAs has significant antifungal activity versus *P. chibensis, B. amyloliquefaciens,* and *P. agglomerans*. The organic (lactic and acetic acid) and phenolic acids (benzoic acid and PLA, among others) produced by BCAs showed antifungal activity against *Fusarium* spp., *A. flavus, Penicillium verrucosum*, and *Alternaria alternata*.

Furthermore, the use of BCAs partially reduced the presence of AFB_1_ and OTA, two mycotoxins of great concern to animal and human health that have been declared carcinogenic to humans. Specifically, the biocontrol strain of *P. polymyxa* CECT 155 decreased the in vitro incidence of AFB_1_ and OTA up to 55% and 20%, respectively. The reduction of mycotoxins production is mainly due to the inhibition of fungal proliferation by the bioactive compounds produced during the fermentation process by BCAs. The present work suggests that both the studied BCA strains and their metabolism products, due to their potential to bring down the growth of the toxigenic fungi and the production of the mycotoxins, could be promising for the bioconservation of food. These strains could be used in combination with starters in fermented foods, and the fermented freeze-dried solution could be used in both formulation and coating of foods. Therefore, these results provide new knowledge for the biotechnological use of BCAs, since they could increase the postharvest shelf-life of food, contributing to meet the demand of consumers, reducing the agricultural use of chemicals and increasing natural alternatives. 

Further investigations are needed to clear up whether the efficacy of BCAs might be enhanced by adding more ingredients and other bio-products that can have synergistic effects.

Finally, the application of BCAs for the biocontrol of mycotoxins should be implemented combined with suitable farming practices and appropriate postharvest management.

## 4. Materials and Methods

### 4.1. Chemicals and Reagents

AFB_1_, OTA, p-coumaric, hydroxycinnamic acid, benzoic acid, DL-3-phenyllactic acid, 1,2-dihydroxybenzene, and 3,4-dihydroxyhydrocinnamic acid were obtained from Sigma-Aldrich (St. Louis, MO, USA). Phenyllactic acid (PLA) was provided from BaChem (Weil am Rhein, Germany). Protocatechuic acid was acquired from HWI Pharma Services (Ruelzheim, Germany). AFB_1_ and OTA had a purity above 99%, while the rest of the analytes had a purity of 95%. Culture media potato dextrose broth (PDB), potato dextrose agar (PDA), plate count agar (PCA), nutrient broth (NB), and triptone soy broth (TSB) were achieved from Liofilchem Bacteriology Products (Teramo, Italy). 

Ethyl acetate, methanol, acetonitrile (ACN), and formic acid (analytical grade, purity > 98%) were obtained from Fisher Scientific (Hudson, NH, USA). C18, ammonium formate, magnesium sulfate (MgSO4), and sodium chloride (NaCl) were obtained from Sigma-Aldrich (St. Louis, MO, USA). Deionized water used (<18 MΩ/cm resistivity) was produced with a Milli-Q purification system (Millipore Corp., Bedford, MA, USA).

### 4.2. Microbiological Culture

The strains of *Penicillium verrucosum* CECT 2913 and *Botytis cinerea* CECT 20973 were obtained from the Spanish Type Culture Collection (Science Park of the University of Valencia, Paterna, València, Spain), which is the public reference center for microbial resources in Spain. The strains of *Aspergillus flavus* ITEM 8111, *Alternaria alternata* ITEM 8121, and six *Fusarium strains* (*Fusarium graminearum* ITEM 126, *Fusarium poae* ITEM 9151, *Fusarium langsethiae* ITEM 11031, *Fusarium verticillioides* ITEM 12043, *Fusarium proliferatum* ITEM 12072, and *Fusarium sporotrichioides* ITEM 12168) were obtained from the Agro-Food Microbial Culture Collection of the Institute of Sciences and Food Production (ISPA, Bari, Italy). These fungi were cryopreserved in sterile liquid medium PDB with 30% glycerol at −80 °C. Prior to antifungal studies on solid and liquid medium, the strains were defrosted and grown in solid medium PDA at 25 °C, with periodic transfers to new PDA plates. These pathogens were selected to cover a representative sample of fungi of the genera *Aspergillus*, *Penicillium*, *Fusarium,* and *Botrytis*.

A total of twenty biocontrol strains: *Paenibacillus polymyxa* CECT 153, *Paenibacillus polymyxa* CECT 155, *Bacillus thuringiensis* CECT 197, *Paenibacillus chibensis* CECT 375, *Paenibacillus alvei* CECT 2, *Bacillus licheniformis* CECT 20, *Bacillus megaterium* CECT 44, *Bacillus subtilis* CECT 499, *Bacillus amyloliquefaciens* CECT 493, *Pantoea agglomerans* CECT 850, *Streptomyces griseus* CECT 3276, *Streptomyces calvus* CECT 3271, *Pseudomonas syringae* CECT 312, *Pseudomonas syringae* CECT 4390, *Pseudomonas syringae* CECT 4393, *Candida sake* CECT 1044, *Candida sake* CECT 10034, *Candida oleophila* CECT 11891, *Metschnikowia pulcherrima* CECT 1691, and *Metschnikowia pulcherrima* CECT 10408 were also obtained from the Spanish Type Culture Collection (Table 7) and kept in frozen glycerol (30%) stocks at −80 °C until analysis. The antifungal activity of these 20 biocontrol strains was studied against the 10 toxigenic fungi belonging to the genera *Aspergillus*, *Penicillium*, *Fusarium*, and *Alternaria* described above. These biocontrol strains were selected mainly because of the scarce data in the literature, which has focused almost exclusively on the use of LABs.

Prior to the fermentation process, the strains were cultivated in different mediums at different temperatures according to the best conditions for their growth. *P. polymyxa* (CECT 153 and CECT 155)*, B. thuringiensi, P. chibensis, P. alvei, B. megaterium, B. subtilis,* and *P. agglomerans* were inoculated with NB (13 g in 1000 mL of distilled water) at 30 °C, while *B. licheniformis* and *B. amyloliquefaciens* were inoculated in the same medium at 37 °C. *S. griseus* and *S. calvus* were inoculated with yeast extract–malt extract (4 g, malt extract 10 g, glucose 4 g) at 30 °C. *P. syringae* (CECT 312, CECT 4390, and CECT 4393) were inoculated with TSB (15 g of triptone soy broth in 1000 mL of distilled water) at 26 °C and *C. sake* (CECT 1044 and CECT 10034)*, C. oleophila* and *M. pulcherrima* (CECT 1691 and CECT 10408) were inoculated with glucose peptone–yeast extract (40 g of glucose, 5 g of Triptone, 5 g of yeast extract in 1000 mL of distilled water) at 24 °C.

### 4.3. Process of Obtaining Cell-Free Supernatant (CFS)

After their defrost and recovery, the biocontrol strains were cultured for 12 h in the media described in 4.2 until exponential phase growth. Then, the strains were placed in 50 mL of NB liquid medium (final concentration 10^7^ CFU/mL) and maintained at 30 °C for 72 h. After this time, the liquid medium was centrifuged for 10 min at 7000 rpm to obtain CFS. These CFS were frozen for 24 h at −80 °C prior to lyophilization (FreeZone 2.5 L Benchtop Freeze Dry System, Labconco, Kansas City, MO, USA) and then stored at −19 °C [16,19].

### 4.4. Qualitative Antifungal Activity of Biocontrol Strains

The effect of the BCAs on the growth inhibition of different fungi (listed in Table 2) was studied by the agar diffusion method and overlay assay. In the agar diffusion assay, PDA medium plates were inoculated with the 10 fungi described in Section 4.2 and after making 1 cm wells, these were loaded with 50 μL of lyophilized and resuspended CFS at a concentration of 100 g/L. After incubating the plates, the appearance of inhibition halos was observed [14,16]. 

In the overlay assay, the center of the PCA plates was contaminated with a suspension of the different BCA, and after solidification, they were covered with PDA medium previously inoculated with spores of the different toxigenic fungi. After incubating the plates, the appearance of inhibition halos was observed and measured [17,20].

### 4.5. Quantitative Antifungal Activity of Biocontrol Strains

Sterile 96-well microplates were used for this assay as described by Luz et al. (2020) [16]. The objective was to determine the MIC and minimum fungicide concentration (MFC). In the wells, 100 μL of CFS suspended at doses between 0.1 and 100 g/L were deposited together with 100 μL of PDB liquid medium contaminated with 5 × 10^4^ spores/mL of the fungi described in Section 4.2. Microplates were incubated in the dark at 25 °C for 72 h, and MIC was considered as the lowest concentration of CFS at which no fungal growth was visually detected. To determine the MFC, 10 μL from each well with concentrations above the MIC were seeded onto PDA medium plates and incubated (25 °C, 72 h.), and the presence or absence of fungal growth was observed.

### 4.6. Determination of Organic and Phenolic Acids in CFS

A high-performance liquid chromatography (HPLC) system (Agilent 1100 Series HPLC System, Agilent Technologies, Palo Alto, CA, USA) was used to analyze the organic acids. The samples were diluted in distilled water and injected using a Spherisorb S5 ODS2 reversed-phase column (4.6 mm × 250 mm, 5 μm) (Waters Corp., Milford, MA, USA). The mobile phase was acidified water at pH 2.1 (0.6 mL/min). The diode array detector (DAD) was set to a wavelength of 210 nm.

Prior to phenolic acid analysis, interferences in CFS were removed by the QuEChERS method [15], and the resulting extracts were resuspended with 1 mL H_2_O:ACN (90:10 *v*/*v*). For the determination, samples were injected into an Agilent 1200 HPLC system (Agilent Technologies, Santa Clara, CA, USA) with a Gemini C18 column (50 mm × 2 mm, 100 Å, particle size of 3 μm; Phenomenex). The mobile phases consisted of water (A) and ACN (B), both with 0.1% formic acid at 0.3 mL/min with gradient (0 min, 5% B; 30 min, 95% B; 35 min, 5% B). A Q-TOF-MS (6540 Agilent Ultra High-Definition Accurate Mass), with an Agilent Dual Jet Stream electrospray ionization interface (Dual AJS ESI) in negative mode, was used for mass spectrometry (MS) analysis [19,31,32]. 

### 4.7. Antimycotoxigenic Activity of CFS 

The antimycotoxigenic activity of the CFS by biocontrol microorganisms was evaluated. This assay was carried out by using 20 biocontrol strains (previously listed). For each BCAs, sterile 15 mL Falcon tubes were provided with 5 mL of NB contaminated with 1 mg/L of AFB_1_ and 1 mg/L OTA. Then, 50 µL of a NB suspension with recently grown bacteria (48 h ago) were added. To make this NB with the mycotoxins, 1 L of sterile NB was prepared to which the appropriate amounts of mycotoxin standard (Sigma-Aldrich (St. Louis, MO, USA) were added. Tubes were kept in an oven at 30 °C for 48 h while control tubes (0 h) were analyzed immediately after preparation. Finally, tubes were centrifuged at 4000 rpm for 15 min at 4 °C, and supernatants were filtered (0.22 µm), and vialized for mycotoxin determination by LC-MS/MS-QTRAP, as explained below.

### 4.8. Determination of AFB_1_ and OTA by LC-MS/MS Spectrometry

For mycotoxin analysis, the samples were injected into an HPLC coupled to a 3200QTRAP mass spectrometer (Applied Biosystems, Foster City, CA, USA). The column used to separate mycotoxins was a Gemini NX C18 column (150 × 2.0 mm I.D, 3.0 mm, Phenomenex, Palo Alto, CA, USA). The mobile phases consisted of water (A) and ACN (B), both with 0.1% formic acid and 5 mM ammonium formate at 0.25 mL/min with a linear gradient. The ions transitions used for the AFB_1_ and OTA identification and quantification were *m*/*z* 313.1/241.3 and 284.9 (AFB_1_) and *m*/*z* 404.3/102.1 and 358.1 (OTA) [33]. 

### 4.9. Statistical Analysis

Statistical analysis was performed using the InfoStat software version 2008. The assays were realized in triplicates, and the differences between control and treated groups were analyzed by Student’s t-test, while the differences between the groups were analyzed by one-way ANOVA test. The significance levels were set at *p* ≤ 0.05.

## Figures and Tables

**Table 1:** Antifungal activity of twenty isolated biocontrol strains versus pathogenic *Penicillium, Aspergillus, Fusarium,* and *Alternaria* species by overlay assay. Values are expressed as percentage of inhibition after 7 days ± standard deviation. % Inhibition = (Diameter of control − Diameter of agent)/Diameter of control.

	% Inhibition									
Pathogen	*F. sporotrichioides* ITEM 12168	*F. graminearum* ITEM 126	*F. proliferatum* ITEM 12072	*F. poae* ITEM 9151	*F. verticillioides* ITEM 12043	*F. langsethiae* ITEM 11031	*P. verrucosum* CECT 2913	*B. cinerea* CECT 20973	*A. flavus* ITEM 8111	*A. alternata* ITEM 8121
Diameter control (cm)	8.5	6.5	7.4	6.6	5	5.3	1.6	7.7	Nd	5.5
**Biocontrol agents**										
*P. polymixa* CECT 153	Nd	Nd	Nd	8.3 ± 0.2	Nd	8.1	Nd	10.2 ± 0.1	Nd	Nd
*P. polymixa* CECT 155	45.2 ± 0.1	50.5 ± 0.1	48.2 ± 0.1	48.2 ± 0.1	40.3 ± 0.2	46.0 ± 0.2	19 ± 0.2	38.3 ± 0.1	Nd	34.3 ± 0.1
*B. thuringiensis* CECT 197	40.1 ± 0.1	20.3 ± 0.2	20.2 ± 0.2	18.5 ± 0.2	18.2 ± 0.2	25.2 ± 0.3	Nd	18.2 ± 0.2	Nd	15.4 ± 0.2
*P. chibensis* CECT 375	18.2 ± 0.2	17.4 ± 0.2	19.2 ± 0.2	16.2 ± 0.1	16.3 ± 0.1	25.1 ± 0.2	Nd	20.2 ± 0.1	Nd	19.3 ± 0.3
*P. alvei* CECT 2	16.5 ± 0.1	15.4 ± 0.1	16.6 ± 0.1	16.7 ± 0.2	16.2 ± 0.2	19.4 ± 0.2	Nd	16.5 ± 0.4	Nd	13.8 ± 0.2
*B. licheniformis* CECT 20	16.3 ± 0.2	17.5 ± 0.2	18.7 ± 0.2	16.9 ± 0.1	Nd	16.4 ± 0.1	Nd	16.2 ± 0.1	Nd	14.7 ± 0.1
*B. megaterium* CECT 44	16.2 ± 0.1	19.2 ± 0.1	18.5 ± 0.1	11.4 ± 0.3	Nd	18.3 ± 0.2	Nd	20.2 ± 0.3	Nd	16.3 ± 0.3
*B. subtilis* CECT 499	25.8 ± 0.3	20.2 ± 0.3	21.2 ± 0.3	25.2 ± 0.2	Nd	18.3 ± 0.1	Nd	21.5 ± 0.2	Nd	13.9 ± 0.2
*B. amyloliquefaciens* CECT 493	25.3 ± 0.2	19.2 ± 0.2	22.8 ± 0.2	22.2 ± 0.2	20.2 ± 0.1	16.2 ± 0.2	Nd	33.2 ± 0.1	Nd	20.3 ± 0.2
*P. agglomerans* CECT 850	47.4 ± 0.1	40.3 ± 0.1	37.6 ± 0.3	20.3 ± 0.1	21.2 ± 0.1	16.6 ± 0.1	Nd	25.3 ± 0.3	Nd	22.2 ± 0.1
*S. griseus* CECT 3276	25.9 ± 0.3	19.4 ± 0.2	20.5 ± 0.2	22.2 ± 0.2	Nd	Nd	Nd	18.5 ± 0.2	Nd	14.2 ± 0.1
*S. calvus* CECT 3271	19.5 ± 0.1	20.4 ± 0.3	26.4 ± 0.2	30.2 ± 0.2	20.2 ± 0.3	Nd	Nd	16.2 ± 0.1	Nd	11.5 ± 0.2
*P. syringae* CECT 312	22.5 ± 0.2	35.3 ± 0.2	25.3 ± 0.1	21.3 ± 0.1	19.4 ± 0.2	Nd	Nd	16.2 ± 0.2	Nd	16.6 ± 0.3
*P. syringae* CECT 4390	22.1 ± 0.2	21.2 ± 0.2	Nd	Nd	18.6 ± 0.1	Nd	Nd	16.3 ± 0.1	Nd	35.4 ± 0.1
*P. syringae* CECT 4393	20.2 ± 0.2	20.1 ± 0.2	Nd	Nd	17.8 ± 0.1	Nd	Nd	16.1 ± 0.1	Nd	16.0 ± 0.2
*C. sake* CECT 1044	31.2 ± 0.1	40.3 ± 0.1	48.2 ± 0.1	40.3 ± 0.2	20.3 ± 0.2	30.2 ± 0.1	Nd	19.2 ± 0.1	Nd	20.1 ± 0.2
*C. sake* CECT 10034	45.2 ± 0.2	39.3 ± 0.1	45.2 ± 0.2	40.2 ± 0.1	39.2 ± 0.1	30.3 ± 0.1	Nd	33.3 ± 0.1	Nd	33.5 ± 0.1
*C. oleophila* CECT 11891	45.1 ± 0.2	35.2 ± 0.2	45.4 ± 0.1	40.2 ± 0.2	39.2 ± 0.1	35.3 ± 0.2	Nd	20.4 ± 0.3	Nd	21.4 ± 0.2
*M. pulcherrima* CECT 1691	45.2 ± 0.2	40.2 ± 0.1	50.4 ± 0.2	41.3 ± 0.2	40.3 ± 0.1	40.5 ± 0.1	Nd	19.2 ± 0.2	Nd	30.5 ± 0.1
*M. pulcherrima* CECT 10408	45.0 ± 0.2	40.3 ± 0.2	50.1 ± 0.3	40.2 ± 0.1	39.2 ± 0.1	40.2 ± 0.2	20 ± 0.2	40.6 ± 0.4	Nd	20.3 ± 0.3

The experiment was carried out in triplicate (n = 3). Results are expressed as mean ± standard deviation. Abbreviations: Nd: not detected. CECT, Spanish-Type Culture Collection (València, Spain). ITEM, the Agro-Food Microbial Culture Collection of the Institute of Sciences and Food Production (Bari, Italy).

**Table 2:** Antifungal activity of twenty cell-free supernatants (CFS) against *Penicillium, Aspergillus, Fusarium,* and *Alternaria* species by the diffusion agar method. The antifungal activity was expressed as follows: (+) means 8 mm of inhibition zone between the well and fungal growth, (++) means 8–10 mm of inhibition zone between the well and fungal growth, (+++) means > 10 mm of inhibition zone between the well and fungal growth. The well radius was 5 mm.

Pathogen	*F. sporotrichioides* ITEM 12168	*F. graminearum* ITEM 126	*F. proliferatum* ITEM 12072	*F. poae* ITEM 9151	*F. verticillioides* ITEM 12043	*F. langsethiae* ITEM 11031	*P. verrucosum* CECT 2913	*B. cinerea* CECT 20973	*A. flavus* ITEM 8111	*A. alternata* ITEM 8121
**Biocontrol Agents**										
*P. polymixa* CECT 153	-	-	-	-	-	-	-	-	-	-
*P. polymixa* CECT 155	-	-	-	-	-	-	-	-	-	-
*B. thuringiensis* CECT 197	-	-	-	-	-	-	-	-	-	-
*P. chibensis* CECT 375	-	+++	-	+++	+++	+++	+++	-	+++	++
*P. alvei* CECT 2	-	-	-	-	-	-	-	-	-	-
*B. licheniformis* CECT 20	-	-	-	-	+	-	-	-	-	-
*B. megaterium* CECT 44	-	-	-	-	-	-	-	-	-	-
*B. subtilis* CECT 499	-	-	-	-	-	-	-	-	-	-
*B. amyloliquefaciens* CECT 493	-	+++	-	+++	-	-	-	-	-	+
*P. agglomerans* CECT 850	++	+	++	+	+	++	+++	+++	-	+++
*S. griseus* CECT 3276	-	-	-	-	-	-	-	-	-	-
*S. calvus* CECT 3271	-	-	-	-	-	-	-	-	-	-
*P. syringae* CECT 312	-	-	-	-	-	-	-	-	-	-
*P. syringae* CECT 4390	-	-	-	-	-	-	-	-	-	-
*P. syringae* CECT 4393	-	-	-	-	-	-	-	-	-	-
*C. sake* CECT 1044	-	-	-	-	-	-	-	-	-	-
*C. sake* CECT 10034	-	-	-	-	-	-	-	-	-	-
*C. oleophila* CECT 11891	-	-	-	-	-	-	-	-	-	-
*M. pulcherrima* CECT 1691	-	-	-	-	-	-	-	-	-	-
*M. pulcherrima* CECT 10408	-	-	-	-	-	-	-	-	-	-

Abbreviations: CECT, Spanish Type Culture Collection (València, Spain). ITEM, the Agro-Food Microbial Culture Collection of the Institute of Sciences and Food Production (Bari, Italy).

**Table 3:** Minimum Inhibitory Concentration (MIC) and Minimum Fungicidal Concentration (MFC) for three lyophilized CFS fermented by biocontrol strains (PCH375, BA493, and PA850) versus *Penicillium*, *Aspergillus*, *Fusarium,* and *Alternaria* species. The results are expressed as (g/L).

Fungi	Biocontrol Agents					
	*P. chibensis* CECT 375		*B. amyloliquefaciens* CECT 493		*P. agglomerans* CECT 850	
	MIC	MFC	MIC	MFC	MIC	MFC
*F. graminearum* ITEM 126	125	250	Nd	Nd	Nd	Nd
*F. sporotrichioides* ITEM 12168	Nd	Nd	Nd	Nd	Nd	Nd
*F. langsethiae* ITEM 11031	125	250	125	250	125	250
*F. poae* ITEM 9151	125	250	Nd	Nd	125	250
*F. verticillioides* ITEM 12043	125	250	Nd	Nd	125	Nd
*P. verrucosum* CECT 2913	Nd	Nd	Nd	Nd	125	250
*A. alternata* ITEM 8121	Nd	Nd	Nd	Nd	125	250
*A. flavus* ITEM 8111	125	250	Nd	Nd	Nd	Nd

Abbreviations: Nd: not detected. CECT, Spanish Type Culture Collection (València, Spain). ITEM, the Agro-Food Microbial Culture Collection of the Institute of Sciences and Food Production (Bari, Italy).

**Table 4:** Identification and quantification of organic acids (mg/L) produced by the twenty bacteria in CFS. The results are expressed as mean ± standard deviation.

Biocontrol Agents	Organic Acids (mg/L)	
	Lactic Acid	Acetic Acid
*P. polymixa* CECT 153	2622 ± 0.12	1873 ± 0.15
*P. polymixa* CECT 155	3372 ± 0.30	1271 ± 0.12
*B. thuringiensis* CECT 197	3491 ± 0.05	Nd
*P. chibensis* CECT 375	1676 ± 0.01	Nd
*P. alvei* CECT 2	384 ± 0.07	Nd
*B. licheniformis* CECT 20	288 ± 0.04	Nd
*B. megaterium* CECT 44	2350 ± 0.04	Nd
*B. subtilis* CECT 499	2553 ± 0.02	Nd
*B. amyloliquefaciens* CECT 493	1251 ± 0.1	Nd
*P. agglomerans* CECT 850	844 ± 0.1	Nd
*S. griseus* CECT 3276	1327 ± 0.06	Nd
*S. calvus* CECT 3271	1411 ± 0.08	Nd
*P. syringae* CECT 312	788 ± 0.11	415 ± 0.02
*P. syringae* CECT 4390	670 ± 0.06	203 ± 0.01
*P. syringae* CECT 4393	913 ± 0.20	208 ± 0.11
*C. sake* CECT 1044	469 ± 0.06	Nd
*C. sake* CECT 10034	416 ± 0.06	Nd
*C. oleophila* CECT 11891	129 ± 0.01	Nd
*M. pulcherrima* CECT 1691	230 ± 0.01	Nd
*M. pulcherrima* CECT 10408	1675 ± 0.03	Nd

The experiment was carried out in triplicate (n = 3). Results are expressed as mean ± standard deviation. Abbreviations: Nd: not detected. CECT, Spanish Type Culture Collection (València, Spain).

**Table 5:** Identification and quantification of phenolic acids (g/L) produced by bacteria in CFS. The results are expressed as mean ± standard deviation.

Phenolic Acids	Biocontrol Agents								
	*B. thuringensis* CECT 197	*P. polymyxa* CECT 153	*P. chibensis* CECT 375	*B. amyloquefaciens* CECT 493	*B. subtilis* CECT 499	*P. alvei* CECT 2	*B. licheniformis* CECT 20	*B. megaterium* CECT 44	*P. polymyxa* CECT 155
Benzoic acid	0.005 ± 0.03	0.060 ± 0.06	0.023 ± 0.01	0.004 ± 0.02	0.044 ± 0.01	0.067 ± 0.02	0.012 ± 0.02	0.047 ± 0.01	0.062 ± 0.01
DL-3-Phenyllactic acid	0.844 ± 0.06	0.184 ± 0.03	Nd	Nd	Nd	0.195 ± 0.01	Nd	0.058 ± 0.02	0.297 ± 0.02
1-2-Dihydroxybenzene	Nd	Nd	0.040 ± 0.01	0.03 ± 0.02	Nd	Nd	Nd	Nd	Nd
Hydroxicinnamic acid	Nd	Nd	Nd	Nd	Nd	Nd	Nd	Nd	Nd
P-Coumaric acid	0.002 ± 0.01	Nd	Nd	Nd	Nd	Nd	Nd	0.030 ± 0.07	Nd
3-4-Dihydroxyhydrocinnamic	Nd	Nd	Nd	Nd	Nd	Nd	Nd	Nd	0.022 ± 0.06
Protocatechuic acid	Nd	Nd	0.008 ± 0.01	Nd	Nd	Nd	Nd	Nd	Nd

The experiment was carried out in triplicate (n = 3). Results are expressed as mean ± standard deviation; Abbreviations: Nd: not detected. CECT, Spanish Type Culture Collection (València, Spain).

**Table 6:** Antimycotoxigenic activity of twenty biocontrol strains versus aflatoxin B_1_ (AFB_1_) and ochratoxin A (OTA) (1 mg/L) after 48 h of exposure.

Biocontrol Agents	% Reduction AFB_1_	% Reduction OTA
*P. polymixa* CECT 153	11.19 ± 0.9	11.85 ± 4.0
*P. polymixa* CECT 155	55.00 ± 3.1	18.78 ± 11.6
*B. thuringiensis* CECT 197	12.71 ± 0.2	22.06 ± 3.7
*P. chibensis* CECT 375	9.81 ± 4.0	7.33 ± 6.2
*P. alvei* CECT 2	19.19 ± 0.9	22.80 ± 2.4
*B. licheniformis* CECT 20	11.81 ± 7.9	26.50 ± 4.4
*B. megaterium* CECT 44	28.87 ± 24.0	13.10 ± 3.5
*B. subtilis* CECT 499	13.41 ± 6.7	13.67 ± 7.0
*B. amyloliquefaciens* CECT 493	0.00 ± 0.0	10.30 ± 7.7
*P. agglomerans* CECT 850	5.00 ± 5.0	7.26 ± 3.2
*S. griseus* CECT 3276	3.51 ± 2.3	21.38 ± 2.2
*S. calvus* CECT 3271	0.00 ± 0.0	10.96 ± 0.8
*P. syringae* CECT 312	6.15 ± 0.13	16.06 ± 7.5
*P. syringae* CECT 4390	28.09 ± 1.6	13.09 ± 3.8
*P. syringae* CECT 4393	26.33 ± 0.7	4.73 ± 9.2
*C. sake* CECT 1044	1.18 ± 2.2	7.33 ± 3.3
*C. sake* CECT 10034	24.86 ± 5.9	10.07 ± 6.6
*C. oleophila* CECT 11891	0.00 ± 0.0	18.69 ± 3.5
*M. pulcherrima* CECT 1691	0.00 ± 0.0	7.19 ± 3.5
*M. pulcherrima* CECT 10408	0.00 ± 0.0	8.41 ± 4.7

**Table 7:** Information on the origin of strains used as biocontrol agents (available at the website of the Spanish Type Culture Collection).

Biocontrol Agent	CECT ID	Source of Isolation	Year	Country	First Description
*P. polymixa*	153	Water	1981	Unknown	Prazmowski, 1880
*P. polymixa*	155	Decomposing plants and soil	1974	Unknown	Prazmowski, 1880
*B. thuringiensis*	197	*Ephestia kuhniella* (Mediterranean flour moth)	1980	Unknown	Berliner, 1915
*P. chibensis*	375	Unknown	1979	Unknown	Shida et al. 1997
*P. alvei*	2	Foulbrood in bees	1974	Unknown	Cheshire and Cheyne 1885
*B. licheniformis*	20	Unknown	1984	Unknown	Weigmann, 1898
*B. megaterium*	44	Unknown	1980	Unknown	Bary, 1884
*B. subtilis*	499	Unknown	1982	Unknown	Ehrenberg, 1835
*B. amyloliquefaciens*	493	Bacterial amylase HT concentrate	1981	United States	Priest et al. 1987
*P. agglomerans*	850	Knee laceration	1987	Unknown	Ewing and Fife, 1972
*S. griseus*	3276	Soil	1987	United States	Krainsky, 1914
*S. calvus*	3271	Soil	1986	India	Backus et al. 1957
*P. syringae*	312	*Nicotania tabacum*	1978	Hungary	van Hall 1902
*P. syringae*	4390	*Phaseolus vulgaris*	1992	Hungary	van Hall 1902
*P. syringae*	4393	*Lycopersicon esculentum*	1992	United Kingdom	van Hall 1902
*C. sake*	1044	Lambic beer	1985	Belgium	van Uden & H.R. Buckley 1983
*C. sake*	10034	Feces of sheep	1991	Spain	van Uden & H.R. Buckley 1983
*C. oleophila*	11891	Fruit of *Olea europea* (olive)	2001	Unknown	Montrocher, 1967
*M. pulcherrima*	1691	Fruit of *Phoenix dactylifera* (date)	1989	Egypt	Pitt & M.W. Miller 1968
*M. pulcherrima*	10408	Orange	1991	Spain	Pitt & M.W. Miller 1968
